# Evaluating the Reliability of OpenAI’s ChatGPT-4 in Providing Pre-colonoscopy Patient Guidance

**DOI:** 10.7759/cureus.86512

**Published:** 2025-06-21

**Authors:** Akash Patel, Adewale Ajumobi

**Affiliations:** 1 Internal Medicine, Eisenhower Health, Rancho Mirage, USA; 2 Internal Medicine, Riverside Community Hospital, Riverside, USA; 3 Graduate Medical Education, Eisenhower Health, Rancho Mirage, USA; 4 Gastroenterology, Eisenhower Medical Center, Rancho Mirage, USA

**Keywords:** artificial intelligence in medicine, chatgpt, chatgpt-3.5, chatgpt-4, colon cancer awareness, colon cancer prevention, colon cancer screening, large language model, llm, patient education

## Abstract

Background: The integration of artificial intelligence (AI) in healthcare is a growing area of interest. This study aims to evaluate the reliability of OpenAI's ChatGPT-4.0 in providing pre-colonoscopy patient guidance, a critical aspect of gastrointestinal care where patient misconceptions and non-compliance are common challenges.

Methods: The study employed a qualitative design to assess ChatGPT-4.0 against established clinical guidelines from various medical societies. Twenty-five patient-like queries encompassing dietary recommendations, bowel preparation, cardiovascular medications, antibiotic prophylaxis, and diabetes medications management were presented to ChatGPT-4.0. The AI's responses were independently evaluated and classified in terms of their alignment with the guidelines.

Results: ChatGPT-4 demonstrated high accuracy, with all 25 sample queries' responses aligning with the established clinical guidelines. It provided precise guidance on dietary restrictions, medication management, and bowel preparation in accordance with the European Society of Gastrointestinal Endoscopy (ESGE), the U.S. Multi-Society Task Force on Colorectal Cancer (USMSTF), the American College of Gastroenterology-Canadian Association of Gastroenterology (ACG-CAG), the American College of Cardiology-American Heart Association (ACC-AHA), the American Society for Gastrointestinal Endoscopy (ASGE), and the Australian Diabetes Society (ADS).

Conclusion: The high degree of guideline adherence by ChatGPT-4.0 underscores its viability as a dependable resource for patient education. Despite its promising results, the study acknowledges limitations such as the structured nature of patient queries and the lack of real patient interactions. The findings suggest a potential role for AI in augmenting patient education and standardizing information dissemination in healthcare.

## Introduction

The integration of artificial intelligence (AI) into healthcare has attracted growing interest and research over the past decade [1]. AI applications in medicine encompass a broad spectrum, including diagnostic algorithms and tools designed to enhance patient education. AI-driven chatbots have shown potential in enhancing patient communication and education [2]. With the advent of more advanced AI models like OpenAI's ChatGPT-4.0, there is a growing curiosity about their utility in providing medical advice that aligns with established clinical guidelines.

Pre-colonoscopy patient guidance is a critical aspect of gastrointestinal care. Accurate and comprehensible information regarding diet and medication management is essential for the success of the procedure [3]. However, disseminating this information efficiently and effectively remains a challenge, often leading to patient misconceptions and non-compliance [4]. While there has been considerable research on the use of AI in diagnostics and patient care, less is known about its effectiveness in patient education, especially in the context of specific medical procedures like colonoscopy [5].

The primary aim of this study is to assess the degree of concordance between ChatGPT-4.0 responses and clinical guidelines for pre-colonoscopy patient education.

## Materials and methods

Study design

This study utilized a descriptive, guideline-concordance-based design to evaluate the reliability of OpenAI’s ChatGPT-4.0 by comparing its responses to established clinical guidelines for pre-colonoscopy patient guidance. The aim was to determine how well the AI's responses aligned with established clinical guidelines, thus assessing its utility as a potential source of medical advice.

Development of patient-like queries

To evaluate ChatGPT’s reliability in pre-colonoscopy education, 25 simulated patient queries were developed to reflect common concerns encountered in clinical practice. These covered five key domains essential to colonoscopy preparation: dietary recommendations, bowel preparation protocols, cardiovascular medication management (including anticoagulants and antiplatelets), antibiotic prophylaxis, and diabetes medication adjustments. The queries were designed by the first author based on frequently asked patient questions and were reviewed for clinical accuracy and relevance by the senior author, a gastroenterologist with over a decade of experience. The selection of 25 queries was deemed sufficient to ensure a representative sampling across the most relevant and guideline-covered aspects of pre-procedural care.

Interaction with ChatGPT

Each query was presented to ChatGPT 4.0 in a separate session, mimicking distinct patient interactions. The queries were entered between May 22, 2023, and May 30, 2023. This approach was designed to prevent any learning or bias from previous queries, and it ensured that the AI’s response was independent and uninfluenced by prior questions.

Guideline selection for comparative analysis

We utilized guidelines from prominent medical societies such as the European Society of Gastrointestinal Endoscopy (ESGE), the U.S. Multi-Society Task Force on Colorectal Cancer (USMSTF), the American College of Gastroenterology-Canadian Association of Gastroenterology (ACG-CAG), the American College of Cardiology-American Heart Association (ACC-AHA), the American Society for Gastrointestinal Endoscopy (ASGE), and the Australian Diabetes Society (ADS) for comparison. These guidelines are considered benchmarks in their respective areas and offer a comprehensive standard against which the AI's responses were evaluated.

Analysis of ChatGPT responses

Two authors independently evaluated the responses generated by ChatGPT-4.0 and categorized them based on their concordance with established clinical guidelines. Responses were classified into three categories: completely aligned, partially aligned, or not aligned. A response was considered completely aligned if it was fully consistent with the guideline's recommendations, without any omissions or inaccuracies. Partially aligned responses included those that reflected some aspects of the guideline but lacked complete detail or omitted key components. Not aligned responses were those that contradicted or failed to reflect the relevant guideline recommendations. This structured classification approach was implemented to ensure a standardized and unbiased assessment of the AI’s accuracy. Any discrepancies between the two reviewers were resolved through discussion and consensus.

Ethical considerations

Given the use of a publicly accessible AI and the absence of patient-specific data, the study did not require Institutional Review Board (IRB) approval. This aligns with ethical standards for research involving public data sources where no human subjects are directly involved.

Data management and analysis

The responses were systematically logged in Microsoft Excel (Microsoft® Corp., Redmond, WA, USA), which facilitated an organized comparison with the guidelines. For each query-response pair, the corresponding guideline was referenced.

## Results

All 25 responses from ChatGPT-4.0 were categorized as completely aligned with the corresponding established clinical guidelines (Table 1). This unanimous alignment was observed across a diverse range of pre-colonoscopy care topics, affirming the accuracy and reliability of AI-generated medical advice.

**Table 1:** ChatGPT responses to pre-colonoscopy questions ESGE: European Society of Gastrointestinal Endoscopy; USMSTF: U.S. Multi-Society Task Force on Colorectal Cancer; ACG-CAG: American College of Gastroenterology-Canadian Association of Gastroenterology; ASGE: American Society for Gastrointestinal Endoscopy; ADS: Australian Diabetes Society; DOAC: direct oral anticoagulants; ICD: implantable cardioverter defibrillator; IVC: inferior vena cava; NPO: nothing by mouth

Category	Patient-Like Queries	Comparative Guidelines	ChatGPT Recommendation
Dietary recommendations	What diet is recommended on the day before colonoscopy?	ESGE & USMSTF	Recommended low fiber diet, and clear liquid diet. Advised to avoid red fluids.
Bowel preparation	Can I take Gas-X while undergoing bowel preparation for colonoscopy?	ESGE & USMSTF	Didn’t recommend against simethicone
	How should I take my bowel prep regimen for colonoscopy?	ESGE & USMSTF	Recommended split dose regimen
	How do I take bowel cleansing agents for colonoscopy while on a split dose regimen?	ESGE & USMSTF	Recommended taking last half within 5 hours
	When should I take the first dose of split dose bowel regimen before colonoscopy?	ESGE & USMSTF	Start 1st half in the evening before
	When should I take the second dose of split dose bowel regimen before colonoscopy?	ESGE & USMSTF	Last half of bowel prep within 5 hours before
	When should I stop taking any bowel cleansing agent or clear liquid before colonoscopy?	ESGE & USMSTF	Start NPO 2 hours before the procedure
	Can I do a same day bowel prep for an afternoon colonoscopy?	ESGE & USMSTF	Same day bowel prep for noon procedure
Cardiovascular medications	Should I continue or stop my warfarin before my screening colonoscopy?	ACG-CAG	Continue warfarin, recommended against bridging
	Should I continue or stop my Xarelto^®^ before colonoscopy?	ACG-CAG	Recommended DOAC interruptions
	Should I continue or stop my Eliquis^®^ before colonoscopy?	ACG-CAG	Recommended DOAC interruptions
	Should I continue or stop my pradaxa^®^ before colonoscopy?	ACG-CAG	Recommended DOAC interruptions
	I have heart disease, should I continue or stop aspirin before colonoscopy?	ACG-CAG	Recommended continuing the aspirin
	I have heart disease, should I continue or stop plavix^®^ before colonoscopy?	ACG-CAG	Didn’t recommend or opposed drug interruption and deferred to physician.
	I have heart disease, should I continue or stop brilinta^®^ before colonoscopy?	ACG-CAG	Didn’t recommend or opposed drug interruption and deferred to physician.
	I have heart disease, should I continue or stop prasugrel^®^ before colonoscopy?	ACG-CAG	Didn’t recommend or opposed drug interruption and deferred to physician.
	Can I take my blood pressure medications on the day of my colonoscopy?	ASGE	Recommended continuing the blood pressure medications
Antibiotic prophylaxis	I have prosthetic heart valve, do I need antibiotic to prevent heart valve infection before colonoscopy?	ASGE	Recommended against the use of antibiotic prophylaxis
	I have history of heart valve infection, do I need antibiotic to prevent heart valve infection before colonoscopy?	ASGE	Recommended against the use of antibiotic prophylaxis
	I have pacemaker, do I need any antibiotic before colonoscopy?	ASGE	Recommended against the use of antibiotic prophylaxis
	I have ICD, do I need any antibiotic before colonoscopy?	ASGE	Recommended against the use of antibiotic prophylaxis
	I have IVC filter do I need any antibiotic before colonoscopy?	ASGE	Recommended against the use of antibiotic prophylaxis
	I had a knee replacement years ago. Do I need to take any antibiotics before colonoscopy?	ASGE	Recommended against the use of antibiotic prophylaxis
Diabetes medications management	How much insulin should I take on the day of colonoscopy?	ADS	Recommended skipping or taking a reduced dose of insulin
	Can I take my oral diabetes medications on the day of colonoscopy?	ADS	Advised to skip oral antihyperglycemic medication dose

ChatGPT's advice adhered strictly to the ESGE and USMSTF guidelines on diet before colonoscopy, emphasizing the necessity of a low-fiber diet and the avoidance of solid foods the day before the procedure. Questions related to the use of medications like Gas-X and instructions for bowel cleansing agents were accurately addressed by ChatGPT. The AI's responses provided clear, detailed instructions on the preparation process, including the timing and method of bowel cleansing agents according to the ESGE and USMSTF guidelines.

ChatGPT's guidance was fully aligned with ACC-AHA and ASGE guidelines on management of antiplatelets and anticoagulants prior to colonoscopy.

Furthermore, ChatGPT adeptly handled queries regarding antibiotic prophylaxis for patients with various medical devices or conditions, such as prosthetic heart valves and heart valve infections. The responses were in complete concordance with the ASGE guidelines.

ChatGPT's responses to questions about insulin and oral diabetes medication prior to colonoscopy were consistent with guidelines from the ADS.

## Discussion

The growing demand for gastrointestinal procedures, especially among older adults and patients taking complex medications like anticoagulants, creates significant challenges in both pre-procedure preparation and patient education. These challenges generate numerous inquiries from both patients and primary care physicians, underscoring the need for accessible, accurate, and standardized medical guidance [6-9]. In this context, AI-driven tools, particularly large language models (LLMs) such as ChatGPT, have emerged as a potential solution to bridge gaps in patient education and enhance healthcare efficiency [1,2,5]. While ChatGPT has been one of the most widely studied LLMs in the medical field, other advanced models such as Google’s Gemini (formerly Bard), Anthropic’s Claude, DeepSeek, Meta’s LLaMA, and Perplexity AI are rapidly evolving and demonstrating capabilities in generating medically relevant information.

Our study systematically evaluated the reliability of OpenAI’s ChatGPT-4.0 in providing pre-colonoscopy guidance and found a high degree of concordance between AI-generated responses and established clinical guidelines. ChatGPT-4.0 delivered accurate recommendations across key domains, including dietary restrictions, bowel preparation, anticoagulation management, and medication use, reinforcing its potential role in clinical practice [3,4]. These findings align with the growing body of research suggesting that AI-driven models can enhance medical education, patient compliance, and procedural outcomes when effectively integrated into clinical workflows [10,11].

LLMs such as ChatGPT have demonstrated promise in patient education by offering structured, guideline-adherent recommendations. The ability of ChatGPT-4.0 to generate responses that are both medically accurate and comprehensible to patients highlights its potential as a valuable adjunct for gastroenterologists and primary care physicians in improving pre-colonoscopy preparation. Previous studies indicate that gastroenterologists overwhelmingly recognize ChatGPT’s reliability, with 80% agreeing that it provides accurate information, 90% believing it can reduce medication errors, and 100% acknowledging its potential in streamlining patient education [12-14].

Despite these strengths, LLMs also present inherent challenges. A primary concern is the phenomenon of AI “hallucination,” wherein models may generate factually incorrect, exaggerated, or misleading information. In some cases, responses may include over-explaining, unnecessary hyperbole, or confidently presented inaccuracies, particularly when prompts are vague or ambiguous. These issues arise from the probabilistic nature of language generation and limitations in training methodologies. While refining training datasets and implementing real-world clinical validation are critical strategies to mitigate these risks, another essential factor is prompt design. Research has shown that well-structured, specific, and contextually framed prompts can significantly improve the accuracy and relevance of AI-generated responses. Therefore, clinicians and researchers using LLMs must be equipped with a basic understanding of effective prompt engineering to maximize the utility of these tools while minimizing misinformation. Although our study did not observe significant hallucinations in ChatGPT-4.0’s responses, responsible use requires ongoing vigilance and user education as AI technologies continue to evolve [12,15].

Several additional limitations warrant consideration. First, our study employed a structured set of queries, which may not fully capture the diverse and nuanced ways in which real patients phrase their questions. Second, while ChatGPT-4.0 demonstrated high accuracy, it does not provide individualized medical advice, as it lacks access to patient-specific factors such as comorbidities, previous procedures, and personal health history. These constraints highlight the need for AI applications to complement, rather than replace, clinician-patient interactions.

Another critical concern is data privacy and regulatory compliance. Prior studies report that only 10% of gastroenterologists express confidence in ChatGPT’s ability to uphold patient confidentiality and comply with privacy regulations, underscoring significant security concerns [12]. These concerns align with broader debates on AI implementation in healthcare, emphasizing the necessity for stringent oversight to ensure adherence to regulatory standards such as the Health Insurance Portability and Accountability Act (HIPAA).

While AI holds considerable potential to enhance patient education and workflow efficiency, its role should be that of a complementary tool rather than a replacement for human clinicians. Many physicians have voiced concerns that over-reliance on AI could erode the physician-patient relationship, reinforcing the importance of maintaining human oversight in patient interactions [12]. AI models should be deployed to augment clinician decision-making rather than supplant it, ensuring that patient care remains grounded in empathy, critical thinking, and professional expertise.

Our study did not compare ChatGPT-4.0 with other AI tools, such as DeepSeek, Gemini/Bard, Claude, or LLaMA, which have demonstrated advancements in AI-driven medical applications. While previous research suggests that ChatGPT-4.0 outperforms both its predecessor (ChatGPT-3.5) and Google’s Bard in accuracy and coherence [16,17], the rapid evolution of AI necessitates continuous reassessment of these models’ effectiveness and safety in clinical settings.

Moving forward, the integration of AI into gastroenterology should prioritize improvements in data privacy, response accuracy, and the long-term impact of AI-assisted patient education on clinical outcomes. Future research should evaluate AI’s efficacy in real-world patient interactions, particularly its ability to dynamically respond to diverse patient concerns. Additionally, further studies are needed to validate AI-driven guidance across varied patient populations and healthcare settings.

Furthermore, the absence of patient-specific context in ChatGPT’s responses presents a significant limitation in clinical workflows. Real-world decision-making often requires consideration of factors such as comorbid conditions, medication interactions, prior procedural outcomes, and social determinants of health details that AI models currently cannot assess. As a result, when discrepancies arise between AI-generated suggestions and individualized clinical judgment, physicians should prioritize tailored decision-making. Clinicians are advised to interpret AI outputs as preliminary guidance that must be contextualized within the broader clinical picture. Establishing clear protocols for reviewing AI recommendations, incorporating human oversight, and fostering patient communication will be essential to safely integrating such tools into routine care.

As AI continues to reshape medical practice, its implementation should be guided by the principles of accuracy, security, and patient-centered care. Ongoing evaluation and refinement will be essential to ensuring that AI technologies enhance, rather than undermine, clinical decision-making and physician-patient relationships.

## Conclusions

This study shows that ChatGPT-4.0 provides pre-colonoscopy guidance fully aligned with established clinical guidelines from multiple medical societies. This consistency highlights its potential as a reliable tool for standardized patient education, improving information dissemination, and reducing the workload of healthcare providers.

## References

[1] Alharbi MT, Almutiq MM (2022). Prediction of dental implants using machine learning algorithms. J Healthc Eng.

[2] Abd-Alrazaq AA, Alajlani M, Ali N, Denecke K, Bewick BM, Househ M (2021). Perceptions and opinions of patients about mental health chatbots: scoping review. J Med Internet Res.

[3] Doykov D, Andonov V (2019). Risk factors and incidence of poor bowel preparation in elderly patients: prospective study. Folia Med (Plovdiv).

[4] Jones RM, Woolf SH, Cunningham TD, Johnson RE, Krist AH, Rothemich SF, Vernon SW (2010). The relative importance of patient-reported barriers to colorectal cancer screening. Am J Prev Med.

[5] Nagam VM (2023). Diagnostic medical artificial intelligence: futuristic prospects for implementation in healthcare settings. Front Artif Intell.

[6] Acosta RD, Abraham NS, Chandrasekhara V (2016). The management of antithrombotic agents for patients undergoing GI endoscopy. Gastrointest Endosc.

[7] Maida M, Sferrazza S, Maida C (2020). Management of antiplatelet or anticoagulant therapy in endoscopy: a review of literature. World J Gastrointest Endosc.

[8] Scridon A, Balan AI (2023). Challenges of anticoagulant therapy in atrial fibrillation-focus on gastrointestinal bleeding. Int J Mol Sci.

[9] Veitch AM, Vanbiervliet G, Gershlick AH (2016). Endoscopy in patients on antiplatelet or anticoagulant therapy, including direct oral anticoagulants: British Society of Gastroenterology (BSG) and European Society of Gastrointestinal Endoscopy (ESGE) guidelines. Gut.

[10] Klang E, Sourosh A, Nadkarni GN, Sharif K, Lahat A (2023). Evaluating the role of ChatGPT in gastroenterology: a comprehensive systematic review of applications, benefits, and limitations. Therap Adv Gastroenterol.

[11] Bekbolatova M, Mayer J, Ong CW, Toma M (2024). Transformative potential of AI in healthcare: definitions, applications, and navigating the ethical landscape and public perspectives. Healthcare (Basel).

[12] Malik S, Kharel H, Dahiya DS (2024). Assessing ChatGPT4 with and without retrieval-augmented generation in anticoagulation management for gastrointestinal procedures. Ann Gastroenterol.

[13] Yeo YH, Samaan JS, Ng WH (2023). Assessing the performance of ChatGPT in answering questions regarding cirrhosis and hepatocellular carcinoma. Clin Mol Hepatol.

[14] Xie Y, Seth I, Hunter-Smith DJ, Rozen WM, Ross R, Lee M (2023). Aesthetic surgery advice and counseling from artificial intelligence: a rhinoplasty consultation with ChatGPT. Aesthetic Plast Surg.

[15] Davis R, Eppler M, Ayo-Ajibola O (2023). Evaluating the effectiveness of artificial intelligence-powered large language models application in disseminating appropriate and readable health information in urology. J Urol.

[16] Tariq R, Malik S, Khanna S (2024). Evolving landscape of large language models: an evaluation of ChatGPT and Bard in answering patient queries on colonoscopy. Gastroenterology.

[17] Patil NS, Huang RS, van der Pol CB, Larocque N (2024). Comparative performance of ChatGPT and Bard in a text-based radiology knowledge assessment. Can Assoc Radiol J.

